# Isoporous Polyvinylidene Fluoride Membranes with Selective Skin Layers via a Thermal-Vapor Assisted Phase Separation Method for Industrial Purification Applications

**DOI:** 10.3390/membranes12030250

**Published:** 2022-02-22

**Authors:** Da Han Choi, Sei Kwon, Youngmin Yoo, In-Chul Kim, Hosik Park, You-In Park, Sung Yun Yang, Seung-Eun Nam, Young Hoon Cho

**Affiliations:** 1 Green Carbon Research Center, Chemical Process Division, Korea Institute of Chemical Technology (KRICT), Daejeon 34114, Korea; dada67@krict.re.kr (D.H.C.); sei0424@krict.re.kr (S.K.); ymyoo@krict.re.kr (Y.Y.); ickim@krict.re.kr (I.-C.K.); hspark@krict.re.kr (H.P.); yipark@krict.re.kr (Y.-I.P.); 2 Department of Organic Materials Engineering, Chungnam National University, Daejeon 34134, Korea; 3 Department of Advanced Materials and Chemical Engineering, University of Science & Technology (UST), Daejeon 34113, Korea

**Keywords:** PVDF, phase separation, porous membrane, filter, purification

## Abstract

The membrane filtration process is the most widely used purification process in various industries due to its high separation efficiency, process simplicity, and low cost. Although there is a wide range of membrane products with diverse materials and pore sizes on the market, there is a technological gap between microfiltration and ultrafiltration membranes. Here we developed highly porous polyvinylidene fluoride (PVDF) membranes with a selective skin layer with a pore size range of 20 to 80 nm by using a thermal-vapor assisted phase separation method. Porous and bi-continuous sublayers were generated from spinodal decomposition induced by cooling. The overall membrane structure and pore size changed with the dope composition, while the pore size and thickness of the selective skin layer were effectively controlled by water vapor exposure. The excellent nanoparticle removal efficiencies of the prepared PVDF membranes were confirmed, indicating their potential application in high-level purification processes to remove small trace organic or inorganic impurities from various industrial fluids.

## 1. Introduction

Filtration processes are the most commonly used membrane-based separation processes in diverse industrial applications due to their simplicity and low cost [1,2,3]. Various industrial products, including food, dairy, chemicals, pharmaceuticals, and bio-products, are purified by filtration [4,5,6,7]. In general, porous microfiltration (MF, pore size range of 0.05–1 μm) membranes are used for clarification or sterilization of the process solution to remove unwanted particles or microbes [8,9,10], while ultrafiltration (UF, pore size range of 1–100 nm) membranes are usually used to concentrate high molecular weight products that are larger than the pore size. Various MF and UF membranes with a wide range of pore sizes are available for industrial uses on the market [11,12,13]. However, there is a gap between UF and MF membranes due to the fabrication process, membrane structure, and operation methods. Membranes with a pore size range between MF and UF (i.e., 10–100 nm), so-called tight MF or loose UF membranes, have been developed and used for specific applications to remove trace impurities in feed solutions [14,15]. For example, loose UF membranes with a high flux and pore sizes of 20–80 nm are mainly used as the final purification process in drinking water and ultrapure water (UPW) production due to their high flux and productivity [16,17].

However, UF membranes with large pore sizes are hardly used to purify industrial products dealing with highly concentrated fluids due to severe membrane fouling and product loss during filtration. Since the removal efficiency relies entirely on the selective skin layer, pinholes or defects on the membrane surface can largely reduce the removal efficiency. Additionally, a cross-flow filtration system may increase the capital and operation costs for small-quantity production.

Instead, tight MF membranes with a precise cutoff performance in the range of 5–100 nm are promising in various chemical, electronic, and pharmaceutical industries due to their process simplicity and high reliability [15,18]. They can also be used to remove trace organic or inorganic particles in a process solution to improve process efficiency and product quality [15]. For instance, tight membrane filters have been developed for UPW production for state-of-the-art semiconductor processing [19]. Among the tight MF membranes, virus filters are one of the most successful membrane products in the biopharmaceutical industry. Virus filtration is a crucial and mandatory purification process for biopharmaceutical production from animal cells [20,21]. Specialized membrane filters with an average pore size range of 20–80 nm are needed to eliminate parvoviruses and retroviruses in the biological fluids [22,23]. Although the structures of commercial virus filters vary with the preparation methods of the manufacturers, they exhibit extremely high size-selective separation performances. A virus filter allows for a near 100% permeation of 5–15 nm-sized protein molecules (e.g., monoclonal antibodies), while the virus can be strictly eliminated at over 99.99% (log reduction value, LRV > 4–7) [23,24,25,26,27]. Due to the development of chemical and biological processing techniques and the increasing demands on precise membrane filters with high selectivity and reliability, tight filters with pore size ranges between UF and MF are promising for various industrial purification applications.

Although tight membrane filters have narrow pore-size distributions and sharp cutoff performances, they suffer from a low permeate flux since the reduced pores in an entire symmetric MF membrane significantly increase the mass transport resistance. To improve the permeate flux of tight filters, various membrane structures, such as asymmetric, hourglass, dual-layer, and thin-film coatings, have been developed [28,29,30,31,32,33]. Since several membrane manufacturers have fabricated and commercialized them by modifying conventional MF membrane fabrication conditions (e.g., dope composition and phase separation condition), studies on tight membrane formation have rarely been reported in the literature. There are basic preparation methods for porous membranes, including nonsolvent (NIPS), vapor (VIPS), and thermally induced phase separation (TIPS) methods. The membrane morphologies and properties are quite different in terms of the preparation methods. NIPS-based polyvinylidene fluoride (PVDF) membranes have asymmetric porous structures with a definite skin layer, while VIPS- and TIPS-based membranes show mostly symmetric porous structures. Although the pore size of the skin layer can be easily controlled by using a simple NIPS process, the structure of the sublayer is difficult to control, and macrovoids are generally formed due to fast solvent-nonsolvent exchange phenomena. As a result, NIPS-based PVDF membranes show typically a high flux but a low mechanical stability. By using the TIPS or VIPS technique, mechanically stable and symmetric membranes with narrow pore size distributions can be prepared. On the other hand, they showed a relatively low flux and a large pore size. 

Recently, various studies on membrane formation by combining phase inversion processes, such as high-temperature NIPS, N-TIPS, and V-NIPS, have been performed to design pore structures that improve the physical properties and separation performances of porous membranes [34,35,36,37,38,39,40]. We prepared tight PVDF membranes with a selective layer with a controlled pore size and a highly porous sublayer via a combined phase separation method. Stepwise cooling, vapor exposure (pore control), and coagulation processes were utilized to form a selective skin layer with a controlled pore size and a highly porous sublayer with a bi-continuous structure. Various dope compositions and phase inversion conditions were studied to optimize the filtration performance of PVDF membranes.

## 2. Materials and Methods

### 2.1. Membrane Preparation

Dried PVDF (Solef 1015, Solvay, Brussels, Belgium) powder was dissolved in organic solvents with additives. *N*-methyl-2-pyrrolidone (NMP), *N*,*N*-dimethyl acetamide (DMAc), and triethyl phosphate (TEP) were purchased from Sigma Aldrich Korea and used as organic solvents. Ethylene glycol butyl ether (EGBE, Sigma Aldrich Korea, Seoul, Korea) and polyethylene glycol (PEG, 200 Da, Samchun Chemical, Seoul, Korea) were used as demixing and pore-forming additives. The dope names and their compositions used in this study are summarized in Table 1. The mixture was mechanically stirred for 24 h at 100 °C to prepare the homogeneous dope solutions. After degassing for 24 h in a 100 °C oven, the membrane was cast on a polyethylene terephthalate (PET) film at a thickness of 200 μm. The casted polymer film was directly placed in a constant humidity/temperature chamber and then coagulated in a water bath for complete phase separation. To investigate the effect of water vapor on the membrane properties and filtration performance, a PTE-3 membrane was fabricated under various vapor exposure conditions. The relative humidity (water vapor partial pressure) and vapor exposure time were controlled from 50 to 90% and from 0 to 1 min, respectively. The vapor exposure factor was calculated as the product of the water vapor partial pressure (torr) and the vapor exposure time (s). The prepared membranes were washed and stored in a water bath before use.

### 2.2. Characterization

The viscosity of a dope solution was measured at various temperatures by using a viscometer (Gel Timer DV2T, Brookfield Ametek, Middleborough, MA, USA). Scanning electron microscopy (SEM, Quattro S, Thermo Fisher, Waltham, MA, USA) was used to observe the membrane surface and cross-sectional morphologies. The average pore size and the pore size distributions of the prepared membranes were analyzed using a capillary flow porometer (Porolux 1000, Porometer NV, Nazareth, Belgium) with Porefil (16 mN/m, Alfa Wassemann Inc., West Caldwell, NJ, USA).

### 2.3. Separation Performance Evaluation

The separation performance of the membranes was evaluated using dead-end filtration equipment. The membranes were placed in a filtration cell (Amicon stirred cell, 50 mL) with nonwoven polyethylene support to prevent the compression of membranes. Essentially, the open porous bottom surface of the membrane faced the feed solution (MF mode, tight-side down) without stirring to obtain the filtration performance. First, the pure water flux (*J*_0_) was measured at 1 bar using deionized water and calculated by the Equation (1) below,

(1) $$J\left(LMH/bar\right)=\frac{\Delta V}{\Delta t\cdot A\cdot \Delta p}$$

where ΔV (L) is the permeate volume, Δt is the filtration time, A is the effective membrane area, and Δp is the applied pressure. After pure water filtration, 50 mL of various feed solutions were filtered to evaluate the retention performance of the membranes. Latex bead (5 mg/L, 3000 Series Nanosphere Size Standards, Thermo Fisher Scientific), gold nanoparticles (20 nm, BBI Solutions, Crumlin, UK), and bovine serum albumin (BSA, 1 g/L in pH 7.4 phosphate buffer saline (PBS), Sigma Aldrich) were used as rejection markers. The permeate samples were periodically collected during filtration and analyzed via UV-vis spectroscopy (Specord 210 Plus, Analytik Jena, Jena, Germany). The rejection (R) was calculated by the Equation (2) below,

(2) $$R\left(\%\right)=\left(1-\frac{C_{p}}{C_{o}}\right)\times 100$$

where $C_{p}$ and $C_{o}$ are the permeate and feed concentrations (ppm), respectively. The normalized flux was calculated based on the ratio of the filtration flux (*J*) and the pure water flux (*J*_0_). The 20 nm particle retention of several commercial virus filter products was evaluated at the same filtration condition to compare the filtration performance. Since they have a multilayer (2–3 layers) structure, the single membrane flux was calculated by multiplying the flux by the number of layers. The effect of the filtration direction on the separation performance was also observed by operating in UF mode (the skin layer faces the feed solution) both with and without stirring.

## 3. Results and Discussion

### 3.1. Membrane Fabrication

**Figure 1:**
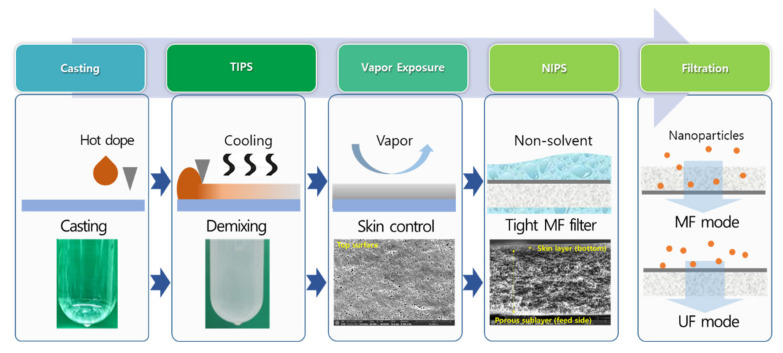
Illustration of the membrane fabrication process and filtration methods used in this study.

The membrane fabrication processes and phase separation steps are illustrated in Figure 1. First, the hot dope solution directly cooled after casting, similar to TIPS, resulting in the spinodal decomposition of casted dope solution before solidification by a nonsolvent (i.e., water). Since heat transport is much faster than mass transport during phase separation processes [41,42], an open porous membrane structure was pre-formed by liquid–liquid demixing [43]. Since organic solvents instead of a diluent for the TIPS process were used in this study, complete solidification was not performed during cooling. In contrast, the spinodal decomposition into polymer-rich clusters and solvent-rich pores was performed at this stage. After casting the dope solution (100 °C), the film of the dope solution was rapidly cooled down to room temperature within 20 s. The casted dope solution was then placed in a chamber with a constant temperature and humidity for a short period of time to control skin layer formation. The delay time (TIPS time) before the vapor exposure was 10 s and the final temperature before the vapor exposure step was 35 ± 3 °C which is close to the chamber temperature. To minimize the vapor contact during TIPS, the temperature and relative humidity of the casting room was set to 22 °C and 30%, respectively. In the chamber, the water vapor induces the phase separation of the membrane, especially the top surface, directly contacting the air. Finally, NIPS was performed for the complete phase separation of the membranes.

**Figure 2:**
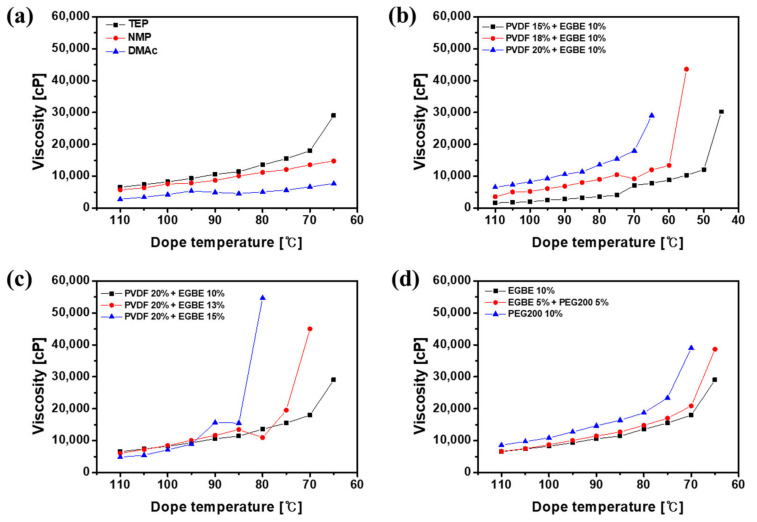
Effects of (**a**) solvent, (**b**) polymer concentration, (**c**) additive concentration, and (**d**) additive ratio on the viscosity and TIPS behavior of dope solutions.

To observe the effects of dope compositions on TIPS phenomena, the viscosities of the prepared dope solutions at different temperatures were measured (Figure 2). Under the initial conditions (100 °C), all of the dope solutions were clear and homogeneous, and the viscosities were below 10,000 cP. As the temperature decreased, the viscosity of the dope solution gradually increased. Notably, the viscosity of the TEP-based dope solution (PTE) jumped at 65 °C, indicating the liquid–liquid demixing in the unstable region of the polymeric solution [44], as shown Figure 2a. The cloud point, the starting temperature of the spinodal decomposition [45], was also visually confirmed (Supplementary Materials Figure S1). Below the cloud point, the viscosity is rapidly increased as the liquid–liquid demixing progresses in the unstable region [46]. On the other hand, TIPS was not observed for the dope solution prepared with polar aprotic solvents, such as NMP and DMAc, which are good solvents for PVDF. 

In addition, the formation of the dense skin layers and macrovoids via fast NIPS phenomena can be found in the cross-sectional morphologies for the dope solutions prepared with NMP and DMAc (Figure S2). At the same time, the TEP-based membrane showed a sponge-like bi-continuous structure formed by liquid–liquid demixing in the unstable region [45]. Here, EGBE and PEG act as pore-forming agents and demixing additive for TIPS. As the polymer and additive concentrations increased (Figure 2b,c), the demixing temperature also increased, which translates to the earlier spinodal decomposition of the dope solution during the membrane fabrication processes. PEG and EGBE showed a similar demixing effect as shown in Figure 2d, while PEG slightly increased the viscosity and demixing temperature due to its high molecular weight and hydrophilicity compared to EGBE. This implies that control of the spinodal decomposition time, the duration of liquid–liquid demixing, and the membrane morphology is possible with the use of additives. The prolonged liquid–liquid demixing of the dope solution before NIPS could improve the homogeneity of the porous structure. As a result, NMP- and DMAc-based membranes showed a very low water flux (not detected) due to the dense structure and thick skin layer with a low porosity formed only by NIPS. In contrast, the water flux of TEP-based membranes with highly porous sublayer and thin skin layer was 125 LMH/bar.

### 3.2. Effects of Additives on the Membrane Properties

**Figure 3:**
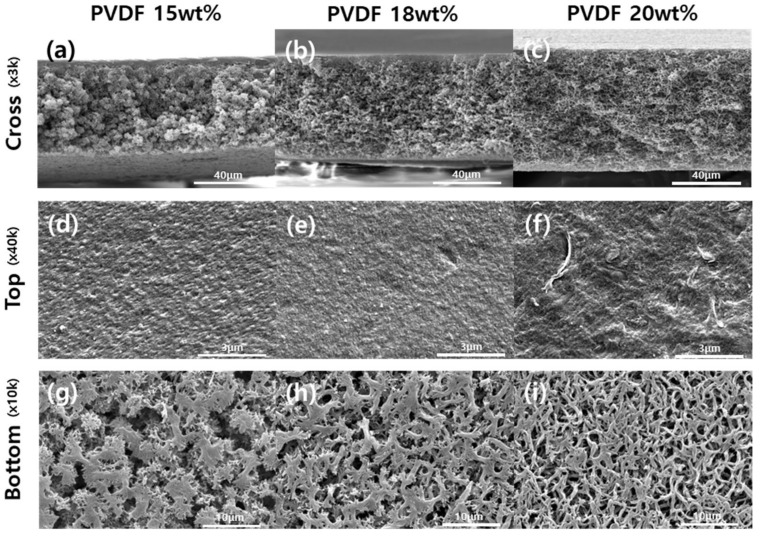
Cross-sectional (**a**–**c**) and surface morphologies (**d**–**i**) of membranes with various polymer concentrations.

**Figure 4:**
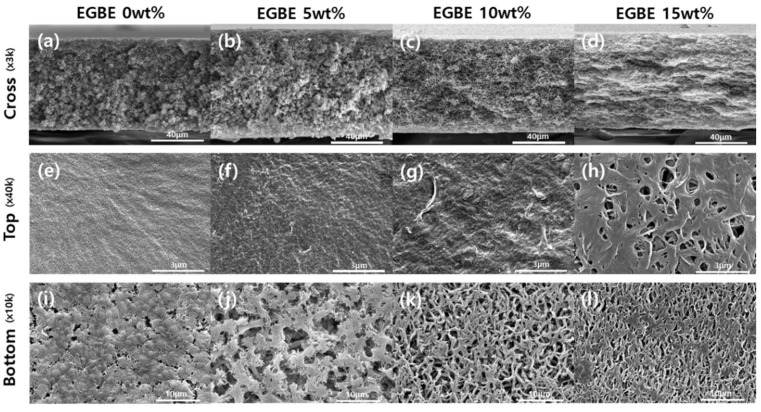
Cross-sectional (**a**–**d**) and surface morphologies (**e**–**l**) of membranes with various EGBE concentrations.

Controlling the dope solution composition affects membrane formation by stepwise phase separation processes, resulting in a change in the separation performance and the overall membrane morphologies and properties. As the PVDF and EGBE concentrations increased, the membrane morphologies changed from spherulitic to sponge-like structures (Figure 3 and Figure 4). Since the dope solution was relatively stable at low polymer and additive concentrations, the demixing of the dope solution were delayed, resulting in the formation and growth of polymer clusters (i.e., spherulites). As the polymer and additive concentrations increased, the spinodal decomposition of the dope was promoted, resulting in limited spherulite growth during phase separation. In addition, the pore size can also vary with the polymer and additive concentrations. In general, the pore size decreases as the polymer concentration increases, while the pore-forming additive can increase the pore size of the membrane. Without the additives, the membrane showed a very low water flux (<1 LMH/bar) due to the dense skin layer (Figure S3). As the EGBE composition in a dope solution increased, the water flux was improved by both demixing and pore-forming effects. At the highest EGBE concentration (15 wt%), the selective skin layer was eliminated, and large pores on the surface were generated, resulting in a high flux (>3000 LMH/bar).

**Figure 5:**
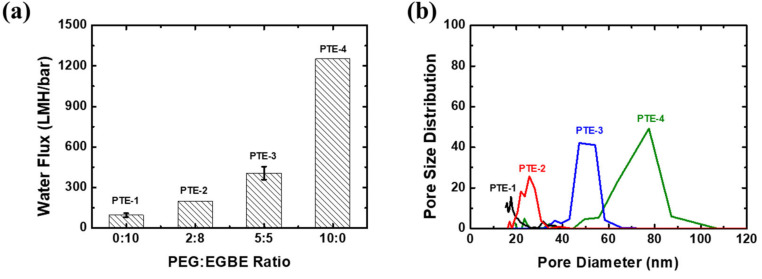
Effects of PEG ratios on (**a**) the water flux and (**b**) the pore size distribution of the PTE membranes.

PEG, which was used as a pore-forming agent in this study, did not change the membrane morphologies much (Figure S4), while the water flux and mean pore size were increased with an increasing PEG ratio as shown in Figure 5. Although both EGBE and PEG had similar effects on the dope solution viscosity, as discussed previously, the pore generation effect of PEG was stronger than that of EGBE due to its high molecular weight and hydrophilicity. The pore size of the selective skin layer gradually increased with an increasing PEG composition, resulting in a dramatic increase in the water flux. When EGBE was substituted by PEG, a highly porous surface was observed with a much higher water flux than EGBE. To conclude, the pore size of the membranes was easily controlled by using pore-forming agents during thermal- and nonsolvent-induced phase separation processes.

### 3.3. Effects of Water Vapor Exposure on the Membrane Properties

The modification of dope composition can change the overall membrane porosity, pore size, and structure. When the polymer concentration was changed, or additives were used to increase or decrease the pore size of the selective skin layer, the phase separation phenomena changed, resulting in a change in the properties of the membrane. The change in the sublayer morphology can be confirmed in Figure 3 and Figure 4 by adjusting the polymer and additive concentrations in the dope solutions. Instead, the pore size and the thickness of the selective skin layer of the membrane were controlled without a change in the sublayer structure by applying the VIPS technique. By supplying water vapor on the membrane surface after membrane casting, the phase separation of the membrane can be performed, especially for the selective skin layer. Although the VIPS technique is often utilized to fabricate homogeneous and symmetric porous membranes by using water-absorbing additives and highly humid conditions, the skin layer is greatly influenced by atmospheric conditions in the case of short vapor exposure. As a result, the pore size of the active layer can be simply controlled by the water vapor partial pressure and exposure time.

**Figure 6:**
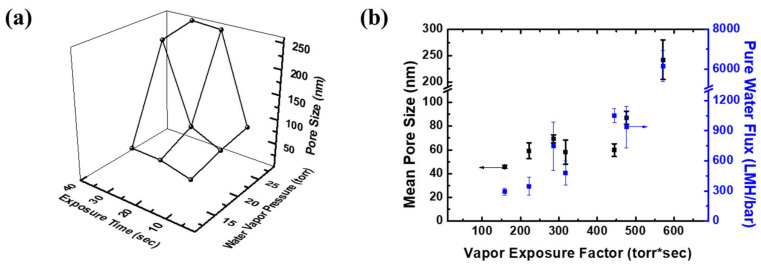
Effects of VIPS conditions on (**a**) the pore size of the selective skin layer of PTE-3 membranes and (**b**) the pore size and pure water flux of the PTE-3 membranes.

For instance, the pore size of the selective layer increases from 43 nm (50%, 10 s) to 60 (50%, 30 s) and 69 nm (90%, 10 s) as the water vapor exposure time and relative humidity increase (Figure 6). In the case of an excessive vapor exposure, the skin layer of the membranes disappeared, and the pore sizes were largely increased above 200 nm, which is the pore size of the sublayer. In addition, the pore size and the water flux of those membranes were at the similar level with commercial microfiltration membranes (i.e., 0.22 μm sterile filters). On the other hand, the overall membrane morphology was not changed by a short vapor exposure, implying that water vapor only affects the surface of the membranes in the dope solutions used in this study. During the VIPS process, the water vapor diffuses through the casted polymer solution, generating a stable pore structure. In a short period of time, water diffusion through the membrane was limited, and only the membrane surface could be tuned by water vapor. As a result, a selective skin layer with a pore size ranging from 20 nm to 80 nm was obtained by a short VIPS process, while the sublayer morphologies were mainly determined by the TIPS and NIPS processes.

**Figure 7:**
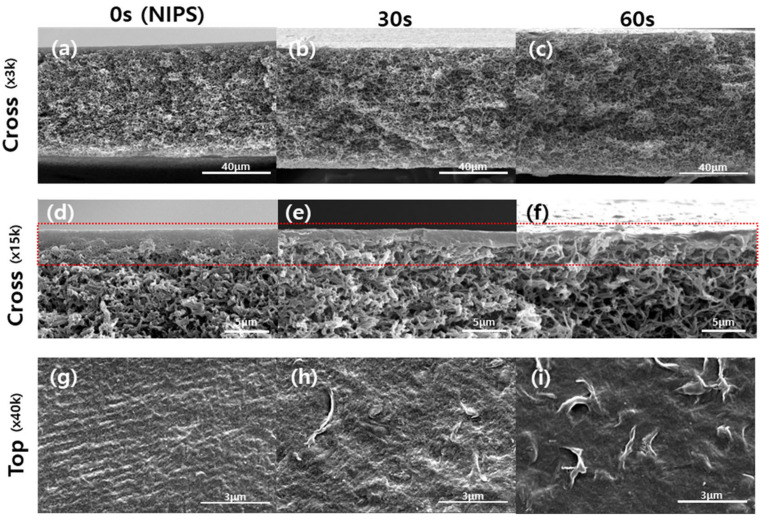
Effects of the vapor exposure time on the morphology of the PTE-3 membrane; Cross-sectional (**a**–**c**), the active layer thickness (**d**–**f**), and the surface (**g**–**i**). Chamber temperature: 30 °C, relative humidity: 50%.

The elimination of the skin layer by the VIPS process can be observed in Figure 7. Initially, the thickness of the selective skin layer was approximately 5 μm without vapor exposure. As the vapor exposure time increased, the thickness of the selective skin layer was reduced, and pore generation on the surface was observed. The same trends were also confirmed by increasing the partial pressure of water vapor (i.e., relative humidity and temperature) (Figures S5 and S6). Therefore, the vapor exposure should be carefully controlled to obtain the selective skin layer with the target pore size.

### 3.4. Filtration Performance

**Figure 8:**
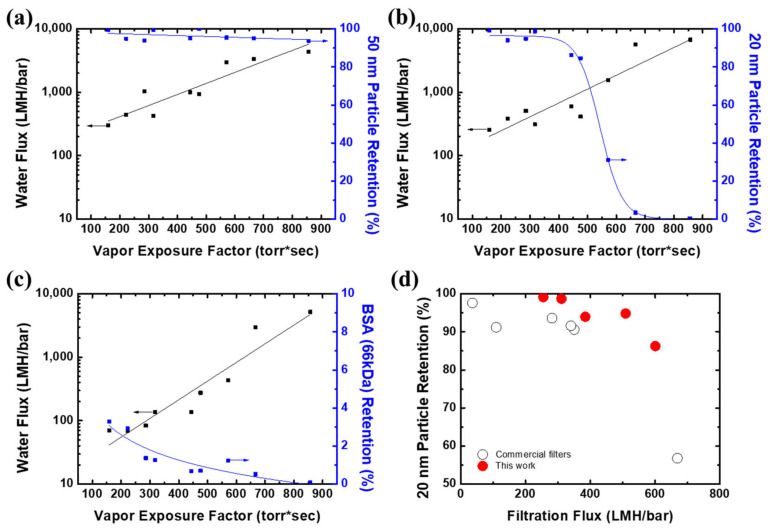
The effect of the vapor exposure factor on the flux and the retention of (**a**) 50 nm particles, (**b**) 20 nm particles, and (**c**) BSA. (**d**) Comparison of the filtration flux and 20 nm particle retention of the PTE-3 membranes with the commercial tight filter products (20–80 nm).

The filtration performance of PTE-3 membranes prepared with different vapor exposure conditions is shown in Figure 8. Since the pore size of the selective skin layer was increased by increasing the vapor exposure factor, the membranes showed a varied purification performance, depending on the vapor exposure factor. For the 50 nm nanoparticles in Figure 8a, the flux was increased and the particle retention was slightly reduced by increasing the vapor exposure factors, while all the membranes showed high retention above 92%. The effect of the vapor exposure factor on the separation performance was more obvious for the 20 nm particles in Figure 8b. The retention was at a high level at a low vapor exposure factor, and it was largely reduced to a vapor exposure factor of 400 due to the enlarged pores at the selective skin layers. For a vapor exposure factor of 600, the membrane allows the permeation of 20 nm particles, while it implies that the efficient separation of 50 and 20 nm particles can be achieved with a membrane prepared with a vapor exposure factor of 600. Likewise, the purification of proteins is possible with the membrane prepared with a vapor exposure factor under 400. The prepared membranes having several tens of nanometers allow for the permeation of BSA, as shown in Figure 8c. Although the flux of tight membranes was largely decreased due to membrane fouling by protein adsorption and pore-clogging, all the membranes showed very low BSA retention properties under 4%. As shown in Figure 8d, the prepared membranes showed higher filtration flux and 20 nm particle retention than the commercial filter products widely used for virus (20–100 nm) filtration in the biopharmaceutical industry. Since the PTE-3 membranes have a highly porous sublayer and thin selective skin layer similarly to thin-film composite membranes, they showed superior flux and retention properties compared to commercial filters, which have dense pore structures [27,47].

From Table 2 it can be seen that the selective skin layer mostly retained the nanoparticles. In MF mode, the nanoparticles penetrated the porous sublayer and were retained by the selective skin layer at the bottom. Au was not detected on the bottom surface, implying that the gold nanoparticles were effectively removed by the membranes. The retention of nanoparticles by both the porous sublayer and the selective skin layer can also be visually confirmed (Figure S7). In MF mode, the filtration efficiency can be improved by the prefiltration effects of the sublayer.

**Figure 9:**
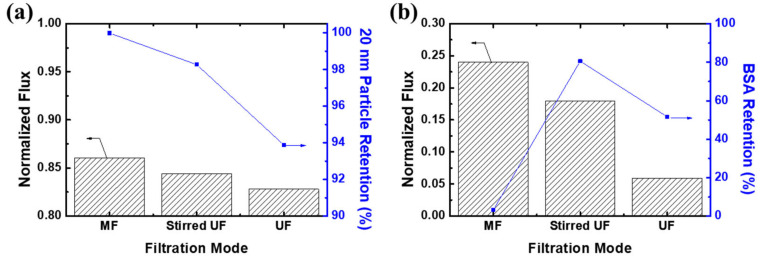
Effect of the filtration mode on the separation performance of PTE-3 membranes. Filtrates: (**a**) 20 nm nanoparticles and (**b**) BSA.

Similarly, the membrane showed a high rejection performance in UF mode, while a small amount of Au was detected in the middle part, indicating leakage of gold nanoparticles in UF mode due to the absence of the prefiltration effect. Figure 9 shows the effect of the filtration mode on the separation performance of the PTE-3 membrane. MF mode showed the highest filtration efficiency among the filtration methods. The particles were successfully retained the PTE-3 membrane in MF mode, while stirred UF and UF modes showed a reduced retention performance. Interestingly, a large portion of BSA was rejected by the PTE-3 membrane in stirred UF mode, even though the pore size was much larger than the size of BSA. Because of the low porosity of the skin layer, BSA could be rejected at the membrane surface under stirring or cross-flow conditions. 

In UF mode without stirring, both the flux and rejection were significantly decreased due to the concentration polarization effect and the cake layer formation on the membrane surface. In conclusion, such a tight filtration membrane can be utilized in both MF and UF applications to expand the scope of membrane separation processes. It was confirmed that the same tight filtration membrane can show different separation performances, depending on the operation mode and conditions. Since they can be used in various purification, recovery, or concentrating applications, the membranes and operation conditions should be decided based on the purpose of the membrane processes.

## 4. Conclusions

Using a thermal-vapor-assisted phase separation process, a highly porous PVDF membrane with a selective skin layer was developed. The prepared dope solution at an elevated temperature was directly cooled, resulting in a bi-continuous sponge-like structure formed by spinodal decomposition, while a selective skin layer was formed on the surface of the membrane via nonsolvent induced phase separation. The membrane’s pore size was controlled by both pore-forming additives and water vapor exposure before coagulation. In particular, the surface pore size was efficiently controlled by water vapor exposure. Prepared PVDF membranes with a pore size ranging from 20 to 80 nm showed efficient filtration performances for nanoparticles and simultaneously allowed the permeation of small protein molecules. Such a tight MF filter can be utilized in various purification processes, including water treatment and diverse chemical and biological industries.

## Figures and Tables

**Table 1:** Dope compositions for the preparation of PVDF membranes.

Dope	PVDF Concentration (wt%)	EGBE Concentration (wt%)	PEG200 Concentration (wt%)	Solvent
PNE	20	10	0	NMP
PDE	20	10	0	DMAc
PTE-0	20	0	0	TEP
PTE-1	20	10	0	TEP
PTE-1A	15	10	0	TEP
PTE-1B	18	10	0	TEP
PTE-1C	20	5	0	TEP
PTE-1D	20	15	0	TEP
PTE-2	20	8	2	TEP
PTE-3	20	5	5	TEP
PTE-4	20	0	10	TEP

**Table 2:** Au concentrations of the top surface, middle part, and bottom surface of the PTE-3 membrane after gold nanoparticle filtration from EDS analysis.

Position	Au Concentration (wt%)	
	MF Mode (Skin = Bottom)	UF Mode (Skin = Top)
Top surface	30.5	93.1
Middle part	7.53	0.38
Bottom surface	0.0	0.0

## Data Availability

Not applicable.

## Supplementary Materials

The following supporting information can be downloaded at: https://www.mdpi.com/article/10.3390/membranes12030250/s1, Figure S1: Photo images of the TEP-based dope solution at different temperatures; Figure S2: Cross-sectional and surface morphologies of membranes (PTE-1, PNE, PDE); Figure S3: Effect of the EGBE concentration on the water flux; Figure S4: Cross-sectional and surface morphologies of PTE membranes with different PEG concentrations; Figure S5: Effects of temperature on the skin layer thickness; Figure S6: Effects of relative humidity on the active layer thickness and the surface morphologies; Figure S7: SEM images of the PTE-3 membrane after 20 nm gold nanoparticle filtration.
