# Supplementary material for: Isoporous Polyvinylidene Fluoride Membranes with Selective Skin Layers via a Thermal-Vapor Assisted Phase Separation Method for Industrial Purification Applications
Source: Membranes (Basel). 2022 Feb 22;12(3):250. doi: 10.3390/membranes12030250 (PMC8953312; doi:10.3390/membranes12030250)
Supplement: Supplementary file 1 [file membranes-12-00250-s001.zip › membranes-1593486-supplementary.pdf]

Supplementary Materials

Isoporous Polyvinylidene Fluoride Membranes with Selective Skin Layers via a Thermal-Vapor Assisted Phase Separation Method for Industrial Purification Applications

Da Han Choi [1,2], Sei Kwon [1], Youngmin Yoo [1], In-Chul Kim [1], Hosik Park [1,3], You-In Park [1], Sung Yun Yang [2,*], Seung-Eun Nam[1,*] and Young Hoon Cho[1,3,*]

[1] Green Carbon Research Center, Chemical Process Division, Korea Institute of Chemical Technology (KRICT), Daejeon, Republic of Korea
[2] Department of Organic Materials Engineering, Chungnam National University, Daejeon, Republic of Korea
[3] Department of Advanced Materials and Chemical Engineering, University of Science & Technology (UST), Daejeon, Republic of Korea
* Correspondence: sungyun@cnu.ac.kr, senam@krict.re.kr, yhcho@krict.re.kr

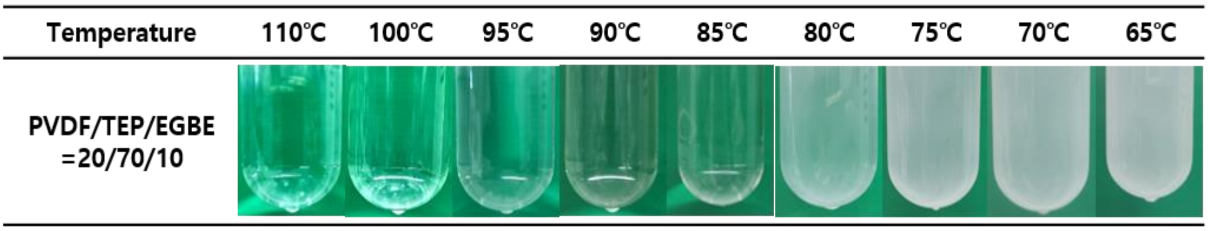

Figure S1. Photo images of the TEP-based dope solution at different temperatures.

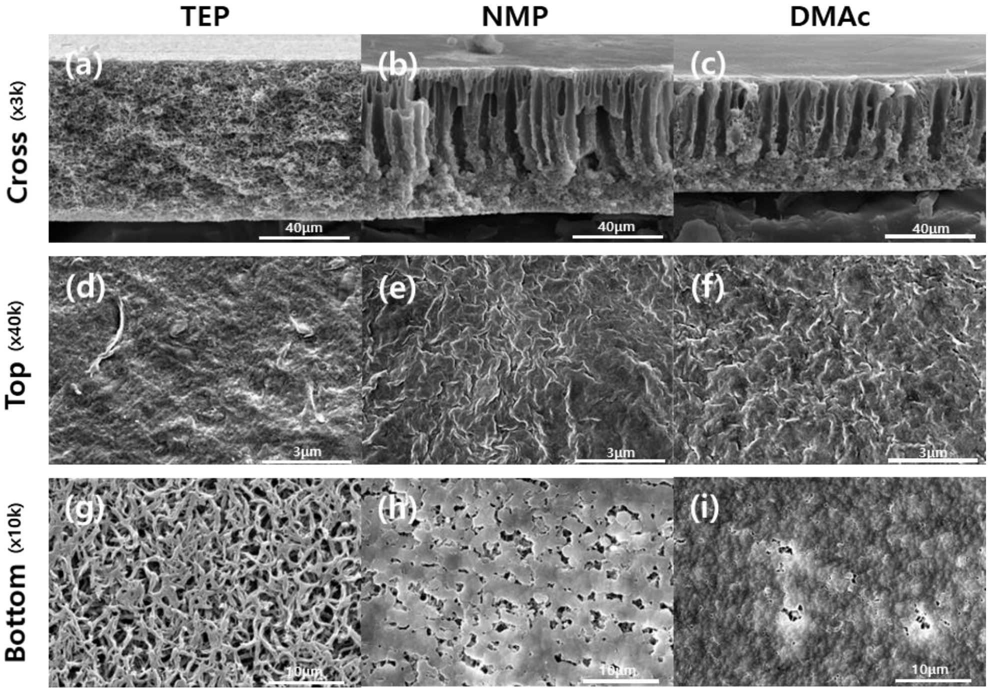

Figure S2. Cross-sectional (a-c) and surface morphologies (d-i) of membranes (PTE-1, PNE, PDE).

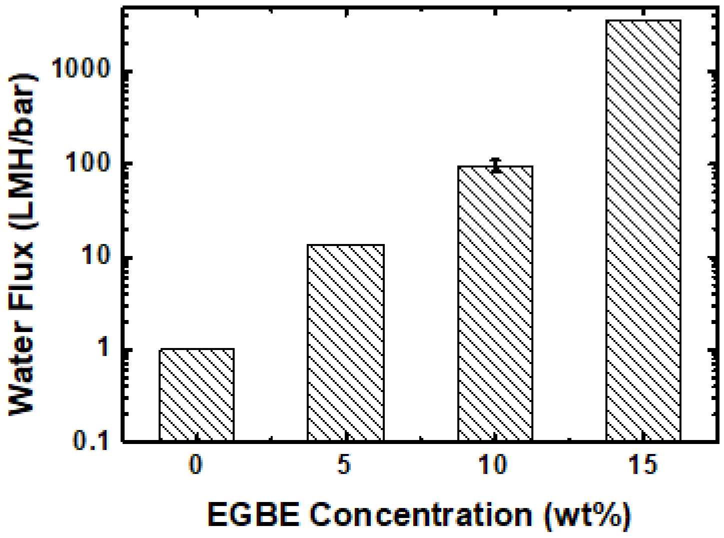

Figure S3. The effect of the EGBE concentration on the water flux.

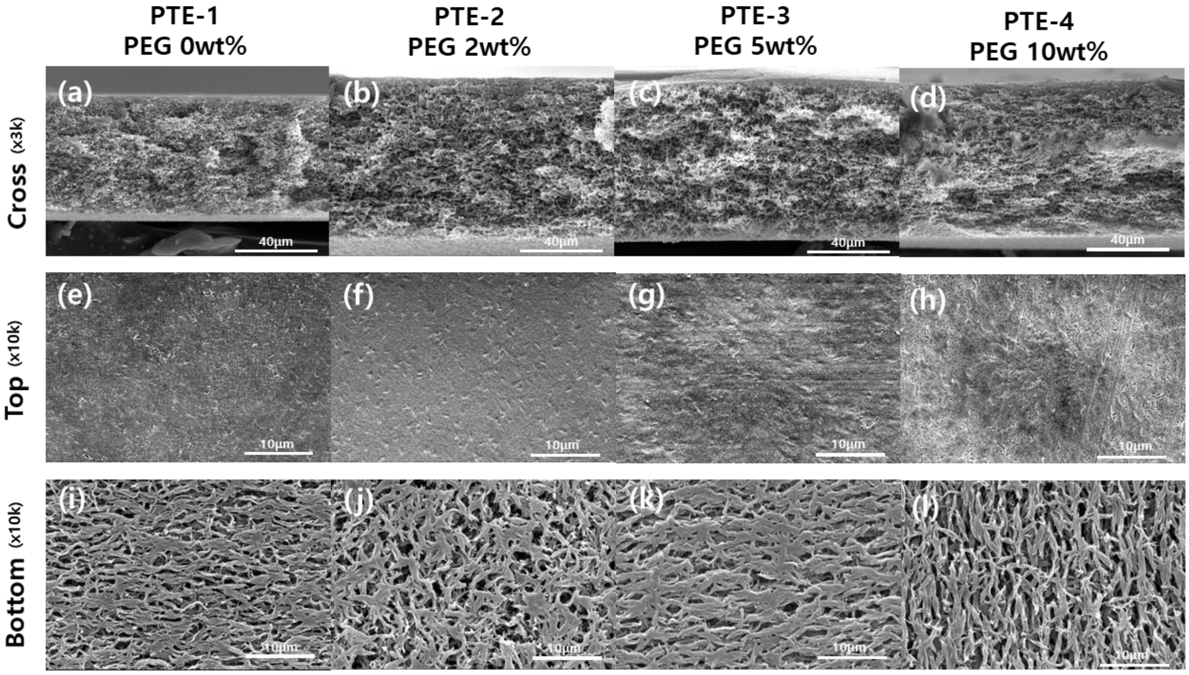

Figure S4. Cross-sectional (a-h) and surface morphologies (e-l) of PTE membranes with different PEG concentrations.

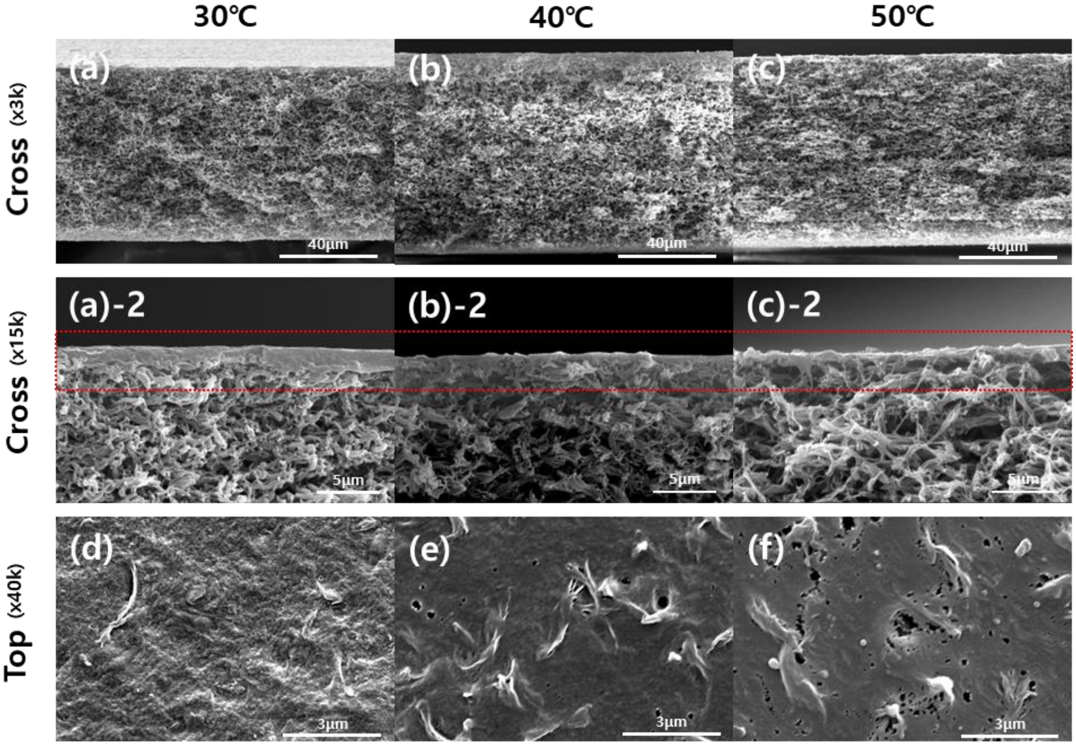

Figure S5. Effects of temperature on the skin layer thickness. Relative humidity: 50%, vapor exposure time: 30 sec.

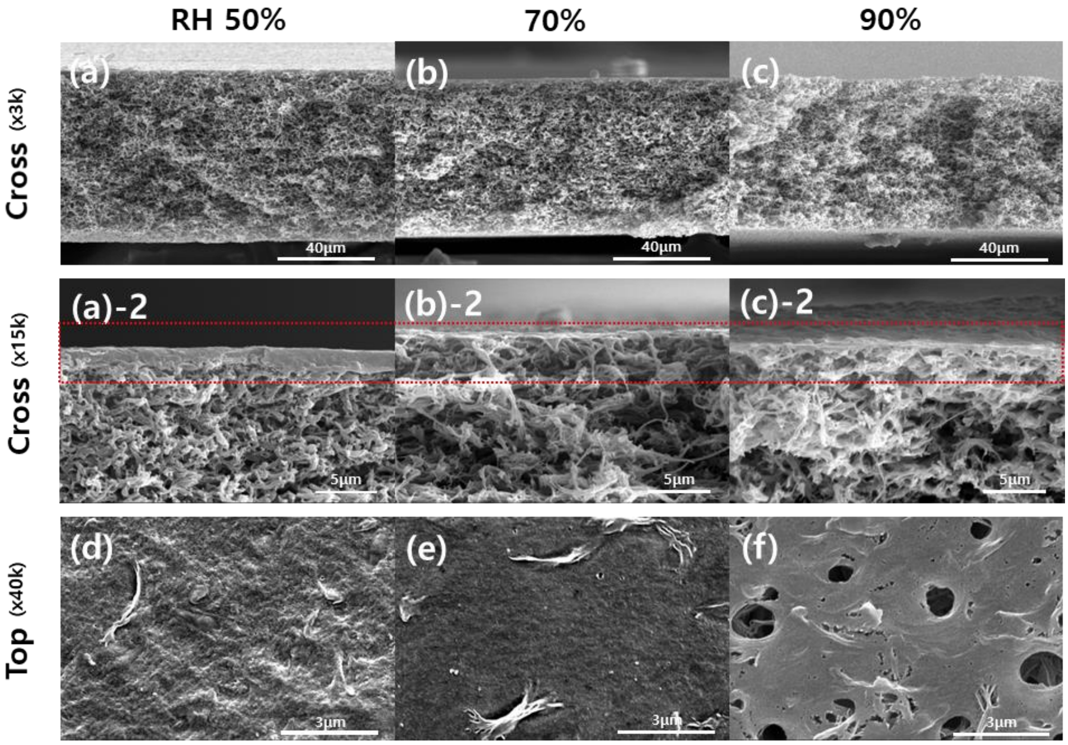

Figure S6. Effects of relative humidity on the active layer thickness and the surface morphologies. Chamber temperature: 30 ºC, vapor exposure time : 30 sec.

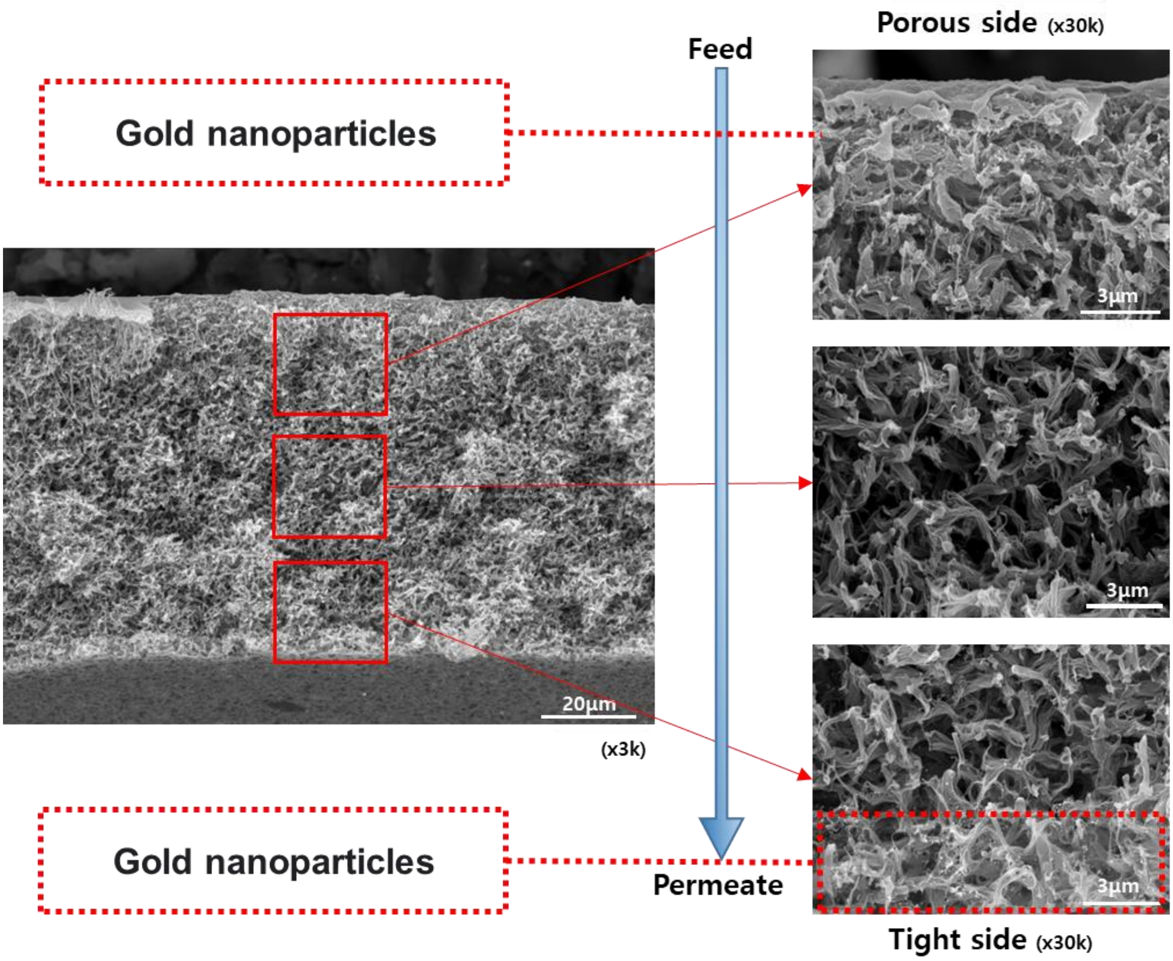

Figure S7. SEM images of the PTE-3 membrane after 20 nm gold nanoparticle filtration.
